# Editorial: Cardioimmunology: Inflammation and Immunity in Cardiovascular Disease

**DOI:** 10.3389/fcvm.2019.00181

**Published:** 2019-12-03

**Authors:** Pietro Enea Lazzerini, Robert Murray Hamilton, Mohamed Boutjdir

**Affiliations:** ^1^Department of Medical Sciences, Surgery and Neurosciences, University of Siena, Siena, Italy; ^2^The Labatt Heart Centre and Translational Medicine, The Hospital for Sick Children and University of Toronto, Toronto, ON, Canada; ^3^VA New York Harbor Healthcare System, SUNY Downstate Medical Center, New York, NY, United States; ^4^NYU School of Medicine, New York, NY, United States

**Keywords:** immunity, inflammation, cardiovascular disease, arrhythmias, heart failure, myocarditis, valve disease

Despite great advances in the diagnosis and treatment witnessed in the last decades, cardiovascular disease (CVD) remains one of the leading causes of morbidity and mortality in Western Countries. This is in part due to the fact that basic pathogenic mechanisms remain in most cases poorly understood, thus significantly limiting the effectiveness of the therapeutic interventions. In this regard, mounting recent evidence shows how immuno-inflammatory activation plays a pivotal role in many cardiovascular disorders, thus opening new unconventional treatment options. Indeed, after the demonstration that atherosclerosis is primarily a chronic inflammatory disease of the arterial wall (1), data suggest that a dysregulation of the immune system and inflammatory pathways may be the leading mechanisms in a large number of CVDs, including heart failure, pericardial disease, cardiomyopaties, and rhythm disorders (2, 3). Immuno-inflammatory mechanisms may play a role in mediating or modulating even hereditary cardiovascular disorders with monogenic etiologies, such as long QT syndrome and arrhythmogenic right ventricular cardiomyopathy (4–6). In the present Frontiers Research Topic, an international selection of investigators contributed original data and up to date reviews to increase our current understanding on the role of the immune system and inflammation in CVD to advance the field forward.

Several contributions focused on the impact of immunity and inflammation on the development of atherosclerosis and related complications. Immune cell trafficking in homeostasis and inflammation is specifically directed and orchestrated by a wide family of chemotactic cytokines, collectively known as chemokines, representing an important protective response toward infectious agents and other injuring factors. However, evidence indicates that excessive or inappropriate activation of the chemokine network is involved in several autoimmune and allergic disorders, transplant rejection, as well as in ischemic heart disease (7). Szentes et al. reviewed the role of the chemokine receptor CXC3 and associated CXC chemokines in the pathogenesis of atherosclerosis and during acute myocardial infarction. They provided evidence that intense chemokine signaling occurs from the forming of the atherosclerotic plaque and plaque destabilization, to all phases of acute coronary events and infarct healing. In this view, they proposed CXCR3-binding chemokines as promising biomarkers for the risk assessment of coronary heart disease, despite the short half-life and the high intra-individual variability. Validation studies in large populations are warranted.

Type 2 immunity, involving specific cell types, such as mast cells, eosinophils, basophils, alternatively activated M2 macrophages, type 2 innate lymphoid cells, and T-helper (Th) 2 cells, and cytokines (IL-4, IL-5, IL-9, IL-13, IL-25, IL-33), and thymic stromal lymphopoietin, are known to critically contribute to the pathogeneses of helminth infection and allergic diseases. However, increasing evidence points to an important role for type 2 immunity actors in maintaining metabolic homeostasis and facilitating the healing process after tissue injury (8). Xu et al. reviewed basic and human data indicating that type 2 immunity-related cell types and cytokines contribute to different physiological and pathological responses after myocardial infarction, particularly by inhibiting the inflammatory activation and promoting angiogenesis and collagen deposition, critical for myocardial repair. Moreover, mast cells can also actively regulate contractility of cardiomyocytes, thereby conferring further potential benefits to the infarcted myocardium. The review provides a framework to deepen our understanding of how type 2 immune responses may facilitate the recovery of cardiac functions after myocardial function, also serving as potential biomarkers for disease severity and prognosis.

Leukotrienes (LTs), acting via several receptors types including BLT1 (receptor for LTB4), participate in various cardiovascular diseases driven by vascular inflammation, particularly atherogenesis and vascular remodeling after angioplasty (9). However, the precise role of BLTR1 signaling in monocytes during vascular inflammation remains unclear. By studying a mouse model of wire-injured femoral artery, Baek et al. provide original data demonstrating that BLTR1 in monocytes is a pivotal player in monocyte-to-macrophage differentiation with subsequent macrophage infiltration into neointima, leading to vascular remodeling after vascular injury. This study adds to our knowledge in the basic mechanisms of vascular inflammation, also supporting the potential role of BLTR1 as an innovative therapeutic target for cardiovascular disease.

Other papers in this Research Topic investigated specific molecular aspects possibly underlying inflammatory pathways involved in the development of CVD. Lysophosphatidylcholine acyltransferase (LPCAT) is a key player in regulating the composition of polyunsaturated phosphatidylcholines (PCs) in mammalian membranes (10). LPCAT3 is highly expressed in macrophages, cells actively involved in atherogenesis in which the plasma membrane provides an important platform that mediates inflammation. Starting from such premises, Jiang et al. demonstrated that in animal models LPCAT3 deficiency promotes membrane PC remodeling and macrophage inflammatory response, specifically increasing toll like receptor-4 expression and inflammatory cytokines release. However, these changes had only a marginal influence on the development of atherosclerosis in mice on a Western type diet.

In another study, Sáez et al. identified the activation of endothelial connexin43 (Cx43) hemichannels as a new pathway affected by inflammatory mediators, supporting a possible involvement of these ion channels in the pathogenesis of CVD. The authors intriguingly proposed that the reduction of hemichannel activity by selective hemichannel blockers might represent a strategy against endothelial dysfunction induced by pro-inflammatory cytokines.

The key role of endothelium in CVD also represented the focus of the paper by Anand et al., by reviewing the impact of human immunodeficiency virus (HIV) on endothelial cells. In fact, cardiovascular events have become an important cause of morbidity and mortality in HIV-infected individuals, where endothelial dysfunction has been identified as a critical link between infection, immune-inflammatory activation, and atherosclerosis (11). By discussing the multiple mechanisms by which viral proteins can damage the vascular endothelium, the authors highlighted how a more detailed exploration into the mechanisms of HIV-induced endothelial dysfunction is essential to develop targeted approaches to prevent and treat HIV-related vascular diseases.

Given the increasingly recognized key role of inflammatory activation in the induction and progression of atherosclerosis, the development of inflammation-targeting therapies as an innovative approach to CVD currently represents a field of great interest. In this view, important lessons derive from rheumatic diseases. In fact, these conditions represent spontaneous “human models” of chronic systemic inflammation associated with accelerated and diffuse atherosclerotic damage, in which anti-inflammatory drugs constitute the cornerstone for patient's treatment (12). By focusing on the main mechanisms linking the inflammatory pathogenic background underlying rheumatic diseases and related vascular damage, Bartoloni et al. analyzed current evidence on the potential atheroprotective effects of disease-modifying anti-rheumatic drugs (DMARDs). Although data suggest that DMARDs, particularly biologic therapies such as TNFα antagonists, may improve surrogate markers of CVD and reduce cardiovascular adverse outcome, the authors highlight as the actual effect of anti-rheumatic therapies on CVD in these patients is rather uncertain due to great literature inconsistency, pointing out to still unresolved questions.

Dipeptidyl peptidase-4 inhibitors (DPP-4i), commonly used as hypoglycemic agents, represent another attractive class of drugs for treating CVD by targeting inflammation. DPP-4 is a protease widely expressed on cell membranes where it plays important roles in immune-regulation, inflammation, and oxidative stress (13). Data indicate that DPP-4i exert potent activities in the cardiovascular system, particularly by regulating blood pressure (BP). Starting from this evidence, Zhang et al. reviewed the current literature and showed that DDP-4i can decrease BP, at least in part by suppressing inflammatory responses and oxidative stress, in turn improving vascular endothelial function. Further research is needed to better define the actual clinical impact of these non-conventional effects of DDP-4i on the cardiovascular system.

The potential contribution of traditional Chinese medicine to the pharmacotherapeutic armamentarium targeting inflammatory pathways involved in cardiovascular damage, is the focus of the study by Zhang et al. These authors investigated the anti-inflammatory activities of ginkolide B (GB), a major monomer extract from leaves of Ginkgo biloba traditionally used in Chinese herbal medicine, during myocardial ischemia/reperfusion (I/R) injury. In fact, inflammatory signaling not only mediates the properties of plaques that precipitate I/R, but also influences the clinical consequences of the post-infarction remodeling and heart failure (14). By using both *in* vivo and *in* vitro I/R models, Zhang et al. provided evidence that GB significantly prevented ultrastructural myocardial changes, also reducing the infarct size. Such beneficial effects resulted from a suppression of the inflammatory response, as demonstrated by the decline in nuclear factor-kappa B activation, inflammatory cytokines expressions, and leukocyte tissue infiltration. Since many patients pay for supplements with claims of benefits, frequently not evidence-based, clinicians who are up to date on the evidence for supplements of traditional Chinese medicine could properly advise their patients on where there may or may not benefit.

Diabetes mellitus (DM) is a progressive metabolic disease characterized by an imbalance in glucose homeostasis, impaired insulin secretion, and abnormal lipid and carbohydrate metabolism. DM represents a major cause of CVD, at least in part by inducing oxidative stress and activating inflammatory pathways in the cardiovascular system. Edible fungi, which contain a number of bioactive components with few adverse effects, are reported to exert many pharmacological effects, including metabolism regulation by reducing the oxidative stress (15). Wang et al. treated diabetic mice with an albino mutant strain of Auricularia cornea and reported significant hypoglycemic effects by reducing blood glucose levels, modulating glucose tolerance, and recovering the serum levels of glycated hemoglobin A1c, glucagon, and insulin. These changes were associated with evident anti-oxidative and anti-inflammatory activities via the regulation of NF-κB signaling. Further research is needed to understand whether natural compounds may have a therapeutic role in reducing the inflammatory burden in diabetes and related cardiovascular involvement.

Heart failure (HF) represents a leading cause of morbidity and mortality in Western countries. Accumulating data in the last few years demonstrated how immune-inflammatory activation is critically involved in the pathogenesis and progression of this condition, with important diagnostic and therapeutic implications (16). Some papers included in this topic further support this view, by both providing new original data and critically reviewing already existing information.

Cardiorespiratory fitness (CRF), defined as the ability of the circulatory, respiratory, and muscular systems to supply oxygen during sustained physical activity, is an objective measure of habitual physical activity and a prognostic indicator in HF (17). Serum levels of C-reactive protein (CRP), a systemic inflammatory marker, and of N-terminal pro-brain natriuretic peptide (NT-proBNP), a biomarker of myocardial strain, also independently associate to adverse outcomes in HF patients (18, 19). In this scenario, van Wezenbeek et al. demonstrated that serum levels of CRP predict CRF impairment in patients with HF across a wide range of ejection fraction, independently from NT-proBNP levels. These new findings point to the inhibition of systemic inflammation with anti-inflammatory drugs as an independent therapeutic strategy improving CRF in patients with HF, thereby adding potential benefits to already existing interventions alleviating myocardial strain.

Obesity is “the disease of the modern era.” It is accompanied by structural and functional alterations in the heart ranging from subclinical impairment of left ventricle systolic and diastolic functions to overt forms of HF (20). Sokolova et al. investigated the involvement of inflammatory pathways in obesity-associated myocardial remodeling, specifically the cytosolic pattern recognition receptor NLRP3, which is an important regulator of the inflammatory cytokine cascade. The evidence that they provided that cardiac concentric remodeling in obesity is modulated by NLRP3 inflammasome, through the effects on systemic inflammation and metabolic disturbances, may open new avenues for preventing HF in obese patients. More research in this fascinating area is warranted.

Systemic inflammation can negatively affect cardiac function and sepsis represents an excellent proof of this concept. In fact, acute HF due to myocardial dysfunction is one of the major complications of severe sepsis, significantly contributing to increased mortality (21). However, the precise underlying mechanisms remain incompletely understood, thereby limiting the development of effective therapies. In a mouse model injected with lipopolysaccharide (LPS), Chen et al. tested the potential benefits of trimetazidine (TMZ), a clinically effective anti-anginal agent which showed protective effects in HF (22). TMZ significantly attenuated cardiac dysfunction, by promoting neutrophil recruitment to cardiac tissue and reducing inflammatory programmed cell death (pyroptosis). Future research is warranted to determine the clinical impact of these intriguing, but yet preliminary data.

Mast cells are ubiquitous innate immune cells chiefly involved in allergic disease and host defense. They act by producing a number of mediators which are also deeply involved in regulating the fibrotic process (23). Legere et al. reviewed current knowledge on the relationship between mast cells and cardiac fibrosis, also underlining how the manipulation of their mediators may represent potential opportunities for intervention. The authors alert us on discrepancies currently existing in the results of both *in-vitro* and animal models, alternatively suggesting mast cells with pro- or anti-fibrotic activities. A better understanding of these findings is urgently needed to move this field forward.

In addition to the unspecific inflammatory activation mediated by the cells of the innate immune system, autoimmune responses of the adaptive immune system to myocardial antigens can contribute to the progression of HF. Starting from recent data demonstrating that autoreactive CD4+-helper T cells specifically targeting cardiomyocytes contribute to the progression of HF (24), Gröschel et al. investigated whether also CD8+-cytotoxic T cells are involved in an animal model of pressure overload-HF induced by transverse aortic constriction (TAC). Although CD8+-cells activate after TAC, this seems to be a largely inefficient process leading only to low-grade cytotoxicity as the progression from cardiac hypertrophy to HF was not significantly accelerated. The authors concluded that, in contrast to CD4+-T cells, CD8+-T cells do not have a major impact on pressure overload-induced HF.

Myocarditis is the archetype of the inflammatory heart disease, resulting from an intricate interplay between microbial agents and immune response, both innate and adaptive (25).

In this Frontiers Topic, Maisch focused on the cardio-immunology of myocarditis, providing an up-to-date review discussing pathogenetic phases and clinical faces of myocarditis, as well as specific treatment options beyond symptomatic HF and anti-arrhythmic therapy. Although great advances in this field have been achieved in the last several years, the author warns the scientific community that there is still much work to be done.

Myocarditis also represents the most common cardiac immune-related adverse event (irAE) during treatment with immune checkpoint inhibitors (ICIs), a new class of monoclonal antibodies, which have shown unprecedented efficacy in treating multiple cancers by promoting the anti-tumor immune response in the host. In fact, activation of immune responses in non-target organs can induce a wide spectrum of irAEs, in some cases also involving the heart. Besides myocarditis, other cardiac irAEs include congestive HF, Takotsubo cardiomyopathy, pericardial disease, arrhythmias, and conduction disease. Tajiri and Ieda reviewed the mechanisms and clinical aspects of cardiotoxicities associated with ICIs, also analyzing available information regarding diagnosis, management, and prognosis. The main message deriving from this review is that although cardiac irAEs are relatively rare, they can be life-threatening, thereby requiring high vigilance from cardiologists and oncologists.

The role of innate lymphoid cells (ILCs) in myocarditis, as well as during cardiac ischemia and healthy conditions, is investigated by Bracamonte-Baran et al. ILCs are a subset of leukocytes with lymphoid properties but lacking antigen specific receptors, considered the link between the innate and adaptive response. The authors demonstrated that the heart, unlike other organs, cannot be infiltrated by circulating ILCs even during cardiac inflammation or ischemia. Thus, the ILCs present during inflammatory conditions, are derived from the heart-resident and quiescent steady-state population, at least in part driven by cardiac fibroblast-derived IL-33 production. If in one hand this study shows that the heart is a unique niche in terms of the ILC compartment, on the other hand it remains to be elucidated at what stage of fetal development or early life, is the heart populated by ILCs.

Accumulating data indicate that the immune system can promote cardiac arrhythmias by means of autoantibodies and/or inflammatory cytokines that directly affect the expression and/or the function of specific ion channels on the surface of cardiomyocytes (26, 27). For these conditions, the terms of autoimmune and inflammatory cardiac channelopathies has recently been coined, respectively (3, 4).

In this topic, Qu et al. comprehensively reviewed the role of autoimmune calcium channelopathies in promoting cardiac rhythm disturbances. They discussed how anti-calcium channel autoantibodies, either inhibitory or agonist-like, are involved in the pathogenesis of the immune-mediated congenital heart block (iCHB), as well as ventricular arrhythmias in patients with dilated cardiomyopathy. Future directions in diagnosis and therapeutic approach are also provided, underlying the potential role of innovative anti-arrhythmic interventions based on the modulation of the immune system or the autoantibody distraction from ion channel binding sites (decoy-peptide based therapy).

Among autoimmune calcium channelopathies, the most investigated is the iCHB. It is a rare but potentially life-threatening rhythm disorders critically related to the transplancental passage of anti-Ro/SSA from the mother to the fetus (28). Ample experimental evidence demonstrated the an inhibitory cross-reactivity of these autoantibodies with the L- and T-type calcium channels plays a key role in the pathogenesis of the disease (29, 30). Fredi et al. provided the first report from the Italian Registry on iCHB, in which 89 cases have been recruited between 1969 and 2017. The paper provided important information regarding pre- and post-natal outcomes, treatment, recurrence rate and maternal follow-up. The authors stated that the registry at present is mainly rheumatological, but the involvement of pediatric cardiologists and gynecologists is planned. Reducing the heterogeneity in management patterns throughout different Italian centers represent the other key point which emerged from this registry.

Aortic valve stenosis, representing the major cardiac valve disease, is characterized by inflammation, atherosclerosis, and calcification (31). Small non-coding RNA (miRNAs) are increasingly recognized as master regulators of gene expression in several physiological and pathological conditions. Specifically, miR-146 is actively involved in the regulation of the immune response as well as in inflammatory process of atherosclerosis (32, 33). Petrkova et al. provided the first report plausibly implicating miR-146a in aortic valve stenosis, thereby indirectly supporting a role for immune-inflammatory activation in the pathogenesis of the disease. More research in this emerging area are warranted.

In conclusion, the high quality contributions of this Research Topic significantly enriched our knowledge of the emerging field of Cardioimmunology, both in terms of basic/translational mechanisms and clinical implications for patients' management. In addition, by emphasizing challenges and unmet needs, this Research Topic provides important directions for further investigation in this fascinating area of cardiovascular medicine and autoimmune and inflammatory diseases.

## Author Contributions

PL contributed to the conception, design and drafting of the work. RH and MB revised the draft. PL, RH, and MB approved the final version and agreed to be accountable for all aspects of the work, ensuring that questions related to the accuracy or integrity of any part of it are appropriately investigated and resolved.

### Conflict of Interest

The authors declare that the research was conducted in the absence of any commercial or financial relationships that could be construed as a potential conflict of interest.
